# Autofluorescence microscopy for paired-matched morphological and molecular identification of individual chigger mites (Acari: Trombiculidae), the vectors of scrub typhus

**DOI:** 10.1371/journal.pone.0193163

**Published:** 2018-03-01

**Authors:** Rawadee Kumlert, Kittipong Chaisiri, Tippawan Anantatat, Alexandr A. Stekolnikov, Serge Morand, Anchana Prasartvit, Benjamin L. Makepeace, Sungsit Sungvornyothin, Daniel H. Paris

**Affiliations:** 1 Faculty of Tropical Medicine, Mahidol University, Ratchathewi, Bangkok, Thailand; 2 Department of Disease Control, Ministry of Public Health, Nonthaburi, Bangkok, Thailand; 3 Department of Infection Biology, Institute of Infection & Global Health, University of Liverpool, Liverpool Science Park IC2, Liverpool, United Kingdom; 4 Mahidol Oxford Tropical Medicine Research Unit, Mahidol University, Bangkok, Thailand; 5 Zoological Institute of the Russian Academy of Sciences, Universitetskaya Embankment 1, Saint Petersburg, Russia; 6 CNRS ISEM—CIRAD ASTRE, Faculty of Veterinary Technology, Kasetsart University, Bangkok, Thailand; 7 Nuffield Department of Medicine, Oxford University, Oxford, United Kingdom; 8 Swiss Tropical and Public Health Institute, Basel, Switzerland; 9 Faculty of Medicine, University Basel, Basel, Switzerland; Chang Gung University, TAIWAN

## Abstract

**Background:**

Conventional gold standard characterization of chigger mites involves chemical preparation procedures (i.e. specimen clearing) for visualization of morphological features, which however contributes to destruction of the arthropod host DNA and any endosymbiont or pathogen DNA harbored within the specimen.

**Methodology/Principal findings:**

In this study, a novel work flow based on autofluorescence microscopy was developed to enable identification of trombiculid mites to the species level on the basis of morphological traits without any special preparation, while preserving the mite DNA for subsequent genotyping. A panel of 16 specifically selected fluorescence microscopy images of mite features from available identification keys served for complete chigger morphological identification to the species level, and was paired with corresponding genotype data. We evaluated and validated this method for paired chigger morphological and genotypic ID using the mitochondrial cytochrome c oxidase subunit I gene (*coi*) in 113 chigger specimens representing 12 species and 7 genera (*Leptotrombidium*, *Ascoschoengastia*, *Gahrliepia*, *Walchia*, *Blankaartia*, *Schoengastia* and *Schoutedenichia*) from the Lao People’s Democratic Republic (Lao PDR) to the species level (complete characterization), and 153 chiggers from 5 genera (*Leptotrombidium*, *Ascoschoengastia*, *Helenicula*, *Schoengastiella* and *Walchia)* from Thailand, Cambodia and Lao PDR to the genus level.

A phylogenetic tree constructed from 77 *coi* gene sequences (approximately 640 bp length, n = 52 new *coi* sequences and n = 25 downloaded from GenBank), demonstrated clear grouping of assigned morphotypes at the genus levels, although evidence of both genetic polymorphism and morphological plasticity was found.

**Conclusions/Significance:**

With this new methodology, we provided the largest collection of characterized *coi* gene sequences for trombiculid mites to date, and almost doubled the number of available characterized *coi* gene sequences with a single study. The ability to provide paired phenotypic-genotypic data is of central importance for future characterization of mites and dissecting the molecular epidemiology of mites transmitting diseases like scrub typhus.

## Introduction

The larval stage of trombiculid mites (family Trombiculidae, subclass Acari) is commonly termed “chiggers” [1, 2]. These larvae are small (approximately 0.2–0.3 mm long) and are recognized vectors of *Orientia tsutsugamushi*, the causative agent of scrub typhus in the Asia-Pacific region. Their bites can result in intense irritation and dermatitis, known as trombidiosis or trombiculiasis in humans and animals, but the frequency and occurrence of this has not been well characterized [3–5]. Taxonomic classification of trombiculid mites is based on exact morphological and morphometric criteria of external characteristics, which have been developed predominantly for the larval stages (usually found during feeding on rodents or other small, terrestrial vertebrates). Unfortunately, detailed classification schemes are highly limited for the soil-dwelling and free-living nymphs and adults [6, 7].

The most important feature for identification and classification of chigger mites is the dorsal shield, termed the scutum (Latin for “shield”). Its shape, size, and distribution of attached setae (fine hairs) and sensilla (sensory hairs) allow assignment of mites to the genus level [8]. Additional characteristics are required for identification to the species level; these include the shape, number and arrangement of idiosomal setae, and features of the gnathosoma and legs [9]. Multiple glossaries of morphological terminology and established identification keys are available, but are mostly based on the larval stage [8–11].

Conventional morphological typing of chigger mites usually requires complex preparation methods to render the specimen sufficiently transparent for microscopic measurements of the scutum and other morphological features. These procedures involve heat and chemical digestion with the use of clearing media (e.g. lactophenol solution, Hoyer’s medium, and Berlese’s fluid) which–despite perfect clarity of the resulting mite preparation–results in total degradation of the mite DNA (and that of any endosymbionts or pathogens contained therein), rendering subsequent genotyping impossible. At present, the entomological investigator must decide to either obtain excellent morphological information or perform robust genetic characterization (typically based on *coi* gene sequencing), but to date, access to both types of data simultaneously from the same individual mite specimen has been difficult to achieve, at best.

Previous attempts involving puncture of the mite at the caudal end to drain a portion of the hemocoel risked jeopardizing morphological findings, and were challenged by difficult DNA extraction procedures due to variation in the available volume and fluctuating DNA yields [12–14]. A common approach has been to work with a large number of chiggers, and to divide these mite specimens derived from the same colonies into batches for either morphological processing or genetic sequencing. Unfortunately, very limited paired and matched data exists for trombiculid mites, despite the need for robust in-depth molecular investigations and prospective characterization of mites.

Aiming to improve mite characterization without any special preparation, we devised a novel approach based on a combination of fluorescence and brightfield microscopy, taking advantage of the autofluorescent properties of the exoskeletons of arthropods. We examined if these autofluorescent properties could be exploited for mite identification following the morphological criteria of previously defined keys for trombiculid mites. As this approach does not require chemical processing of the entire mite, we anticipated that this approach would preserve mite genomic DNA to provide paired-matched morphotyping and genotyping data of the same individual chigger.

## Materials and methods

### Ethics statement

This study was approved by the ethical review committees from National Ethics Committee for Health Research (NHCHR) Lao PDR (No 51/NECHR) and Mahidol University-Institute Animal Care and Use Committee (MU-IACUC) (MU-IACUC 2016/024). All rodent species investigated are not on the Convention on International Trade in Endangered Species of Wild Fauna and Flora (CITES) list, nor the Red List of International Union for Conservation of Nature (IUCN), and were not classified as endangered or protected species. All rodent collection procedures were specifically approved by NHCHR Lao PDR. Animals were treated in accordance with the guidelines of the American Society of Mammalogists, and with the European Union legislation (Directive 86/609/EEC).

### Chigger mite collections

Study sites were selected in Phonehong (Lat 18.28361403, Long 102.512667) and Thulakom districts (Lat 102.535078, Long 231.591385) in Vientiane, Lao PDR. Traps within houses were set with the approval of the owner or tenant. Outdoor traps in agricultural fields or community forests were set with the agreement of the village leader.

Rodents were trapped using wire live-traps (hand-made locally) disposed in lines of 10 traps (every 5 to 10 meters) for three days, with additional captures conducted by locals. Captured rodents were treated in accordance with the guidelines of the American Society of Mammalogists, and with the European Union legislation (Directive 86/609/EEC), and were euthanized using isoflurane [15]. Rodent ears with visible chigger mites were dissected, placed into 70% ethanol, transferred to absolute ethanol ethanol within 6–8 hours, and transported to Mahidol Oxford Tropical Medicine Research Unit (MORU), Bangkok, for imaging under a stereo microscope (Nikon AZ 100, Digital Sight DS-Ri1, and Nikon NIS element software). The obtained image was used to mark chigger mite locations from colonies on the rodent ear, which were selected by purposive sampling for broad representative coverage. Individual chiggers were then picked, according to the location marks on the image, by a fine bristle brush (S1 Fig). A minimum of 7 chiggers per ear were selected for each rodent and stored in absolute ethanol at 4°C. A total of 113 chiggers representing 12 species and 7 genera (*Leptotrombidium*, *Ascoschoengastia*, *Gahrliepia*, *Walchia*, *Blankaartia*, *Schoengastia* and *Schoutedenichia*) from 26 individual rodents were included from a recent Lao PDR field survey dedicated to this methodology development. An additional 153 randomly selected chiggers from previous field surveys in Thailand and Cambodia were included for specific autofluorescence versus conventional microscopy evaluation. The latter specimens were characterized by the classical Berlese clearing method for mite morphology and represented five genera (*Leptotrombidium*, *Ascoschoengastia*, *Helenicula*, *Schoengastiella* and *Walchia*. Some results of these surveys have in part been previously described [16].

### Preparation of chiggers with mounting medium

The Hoyer’s medium preparation method has been used to prepare chigger mites for identification to the species level since the 1950s [17, 18]. This approach required a hot plate, and a small needle to puncture chiggers and remove the internal substances/organs. The exoskeleton was cleared using Hoyer’s medium to provide a transparent preparation that allowed for genus or species classification [1, 18]. Hoyer’s medium contains 50 mL distilled water, 30 g crystalline gum arabic (or gum acacia), 200 g chloral hydrate, and 20 mL glycerin [17].

The Berlese fluid preparation method originally involved soaking chigger mites in deionized water, mounting on slides in the Berlese fluid (based on aqueous gum chloral), followed by microscopic examination and identification via published keys (developed by Professor A. Berlese who did not publish the formula) [1, 18]. In this study, chiggers were soaked in sterile water for at least one hour, mounted dorso-ventrally on glass slides using Berlese fluid (TCS Bioscience Ltd, UK), and covered with round coverslips, exerting gentle external pressure for stretching and flattening of the specimens to increase the accuracy of morphometric measurements. The mounted slides were incubated at 50°C for two days in hot air incubator before further examination [16].

### Chigger examination by fluorescence microscopy

The set of 113 chiggers (Lao PDR study) underwent complete characterization, with imaging for retrospective mite identification to the species level (set of 16 images)—consisting of mouthparts (chelicerae and palpi), legs, ventral and dorsal body and scutum. The set of 153 chiggers from Thailand and Cambodia underwent only scutum imaging with morphometrics, for characterization to the genus level (set of 2 images). Individual mites were aspirated by micropipette and placed between two glass cover slips with sufficient sterile water (preferred) or ethanol (which evaporates rapidly and risks drying out of chiggers), and put on a glass slide for microscopy. No fluorescent dyes were used.

All images were obtained using a fluorescent-isothiocyanate (FITC) single-band filter with an epifluorescence illuminator (mercury or xenon lamps) and/or white light (halogen lamp) in bright-field mode, benefitting from the natural, differential autofluorescent characteristics of the chigger scutum, integument, and appendages. The following combinations were used: epifluorescence with FITC filter for autofluorescence (AF) or a combination of AF with bright-field (AF-BF), while multilayer (ML) images were labeled (ML-AF) or (ML-AF-BF).

Complete characterization images were acquired using a compound microscope (Nikon Eclipse 80i, Nikon NIS element D 4.13.05 software, manual multilayer imaging with CombineZP Image Stacking Software by Alan Hadley). Scutum imaging with morphometric measurements were performed using ZEISS Axio Imager M2 microscope and ZEN 2011 imaging software (Carl ZEISS, Germany).

### Morphological identification

Chiger mite morphological classification begins with subfamily and tribe identification of trombiculid mites, which are identified by the scutum shape and setae alone. Identification at the genus level requires the detailed scutum plus other genus classification characteristics, like specialized leg setae, palpal chaetotaxy, and chelicerae. To follow the identification keys for robust morphotyping to the species level, a large collection of images is required. Identification to the genus level was performed according to identification keys from Nadchatram and Dohany (1974) [8] and Fernandes and Kulkarni (2003) [19]. Identification to the species level utilized identification keys by Stekolnikov (2013) [11], Goff *et al*. (1982) [10], Vercammen-Grandjean and Langston (1975) [9], Traub and Evans (1957) [20], Fernandes and Kulkarni (2003) [19], and Traub and Morrow (1956) [21].

**Fig 1:**
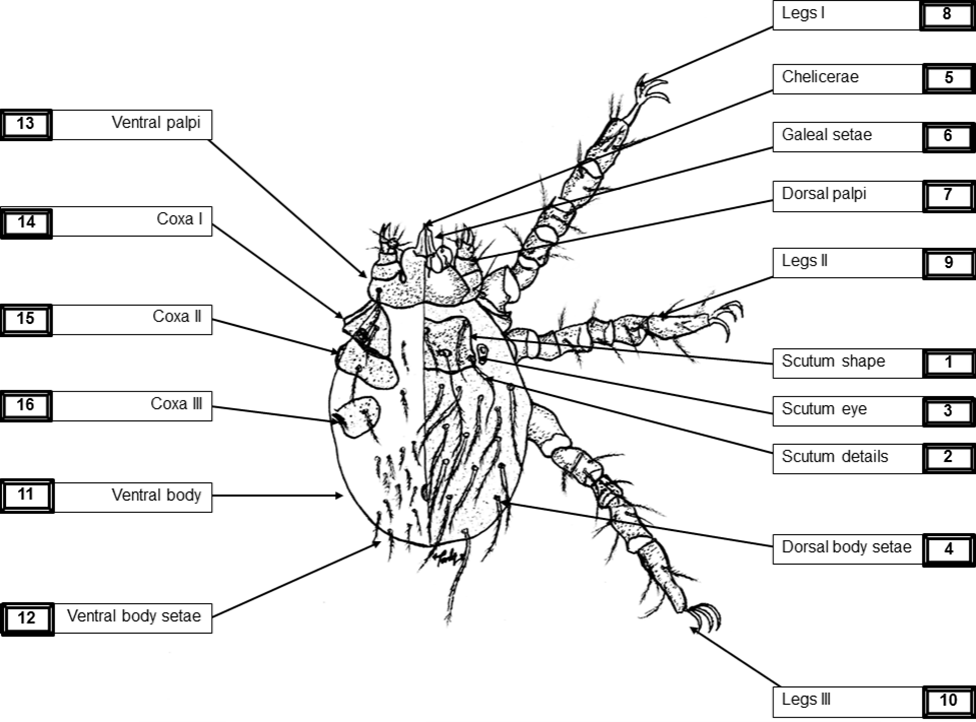
Schematic overview of images required for morphotyping (template panel). A minimum set of 16 defined images are required to retrospectively confirm and differentiate chigger mites to the species level; images; 1 Scutum shape; 2 Scutum details; 3 Scutum eye; 4 Dorsal body setae; 5 Chelicerae; 6 Galeal setae; 7 Dorsal palpi; 8–10 Legs I-III; 11 Ventral body; 12 Ventral body setae; 13 Ventral palpi; 14–16 Coxa I-III. *Note*: *the schematic drawing was prepared by co-author Kittipong Chaisiri*.

All key identifying features of chiggers were determined using AF or BF or a combination of AF-BF, as defined in the checklist (Fig 1 and S1 Table). The data were evaluated and interpreted independently by five entomologists to ensure consensus expert agreement at the genus, subgenus, and species level, where applicable (RK, KC performed primary characterisation; AP, SS and AAS performed independent confirmation of results as senior entomologists).

### Molecular identification

DNA from individual chigger mite was extracted using the QIAGEN Blood & Tissue kit (QIAGEN Sciences, MD, USA) according to manufacturer’s instructions with modifications. Briefly, prior to DNA extraction, ethanol was thoroughly removed by micropipette aspiration and 3 times washing with 50 μl of deionized water. Chiggers were then submerged in tissue-lysis buffer and cut into small pieces with a 30G needle under an inverted microscope. Following the addition of Proteinase K, the sample was incubated at 56°C for 3 hours. Binding buffer and ethanol was added and the suspension was transferred into the spin column. After washing 2 times, the DNA was eluted using the kit’s elution buffer. DNA quantity and purity was assessed using a NanoDrop 2000 Spectophotometer. The extracted DNA was kept at 4°C for immediate PCR assay or at -20°C for long term storage.

PCR assay was used to amplify an approximately 710 bp fragment of the mitochondrial cytochrome c oxidase subunit I gene (*coi*), as previously described [22]. This assay has been evaluated in 11 invertebrate phyla (Arthropoda, Echinodermata, Mollusca, Annelida, Pogonophora, Nemertinea, Echiura, Sipuncula, Platyhelminthes, Tardigrada, and Coelenterata) to provide informative sequences for phylogenetic analyses at the species and higher taxonomic levels. Post-PCR amplicons were visualised on 1.5% agarose gel and purified using a QIAquick® PCR Purification Kit (250) according to manufacturer’s instructions prior to commercial sequencing (Macrogen, Seoul, Korea). All *coi* sequences were base-called, aligned, and cropped to an equal length of 637 bp. Identity percentage of significant alignments were compared by BLAST ^®^ and served to construct a phylogenetic tree (CLC Workbench v7.7.1 and MEGA v6 software).

## Results

### Field surveys

From the Lao PDR field survey, a total of 113 chiggers (comprising 12 species of 7 genera) were characterized to the species level. In total, 71 rodents (comprising 9 rodent species) were caught including 26 rodents that carried chigger colonies in their ears (S1 Fig) (Table 1).

**Table 1:** Summary of rodents and mites collected from the Lao study, with subsequent morphotyping and genotyping.

Rodent species *(N captured)*	Rodents with chiggers	Rodent code	Chigger taxa	Successfully morphotyped	Successfully genotyped
*Bandicota indica (n = 15)*	6/15 (40%)	L0499, L0516	*Blankaartia acuscutellaris*	4	3
		L0522, L0553	*Walchia ewingi ewingi*	2	1
* *		L0556, L0568	*Walchia ewingi lupella*	18	9
* *			*Leptotrombidium deliense*	6	3
* *			*Schoengastia kanhaensis*	3	1
*Berylmys berdmorei (n = 1)*	0/1	*-*	*-*	0	0
*Berylmys bowersi (n = 1)*	1/1	L0563	*Gahrliepia marshi*	1	0
* *			*Gahrliepia tylana*	1	0
* *			*Walchia alpestris*	5	2
*Maxomys surifer (n = 3)*	3/3	L0560, L0561, L0562	*Walchia kritochaeta*	11	4
* *			*Walchia minuscuta*	1	1
* *			*Leptotrombidium deliense*	1	0
*Mus caroli (n = 9)*	3/9 (33%)	L0532, L0533	*Walchia kritochaeta*	1	1
* *		L0538	*Walchia minuscuta*	7	0
*Niviventer sp*. *(n = 4)*	3/4 (75%)	L0565, L0566	*Ascoschoengastia indica*	10	5
* *		L0567	*Walchia kritochaeta*	1	0
* *			*Walchia minuscuta*	2	1
*Rattus exulans (n = 20)*	0/20		* *	0	0
*Rattus sakaretensis (n = 10)*	5/10 (50%)	L0523, L0540, L0542	*Blankaartia acuscutellaris*	7	7
		L0547, L0555	*Walchia ewingi ewingi*	6	1
* *			*Walchia ewingi lupella*	1	0
* *			*Leptotrombidium deliense*	6	2
*Rattus tanezumi (n = 8)*	5/8 (63%)	L0508, L0524	*Ascoschoengastia indica*	10	1
		L0543, L0557	*Schoutedenichia centralkwangtunga*	1	1
* *		L0569	*Walchia kritochaeta*	1	0
* *			*Walchia minuscuta*	3	1
* *			*Leptotrombidium deliense*	4	3
**Total**	**26/71 (37%)**			**113**	**47**

From the field surveys from Thailand (Nan) and Cambodia, a total of 153 mites, previously characterized by the Berlese fluid preparation method using conventional pictorial keys, were available for additional AF-BF analyses of their scuta [16, 23] (S2 Fig).

### Use of FITC filter for imaging

**Fig 2:**
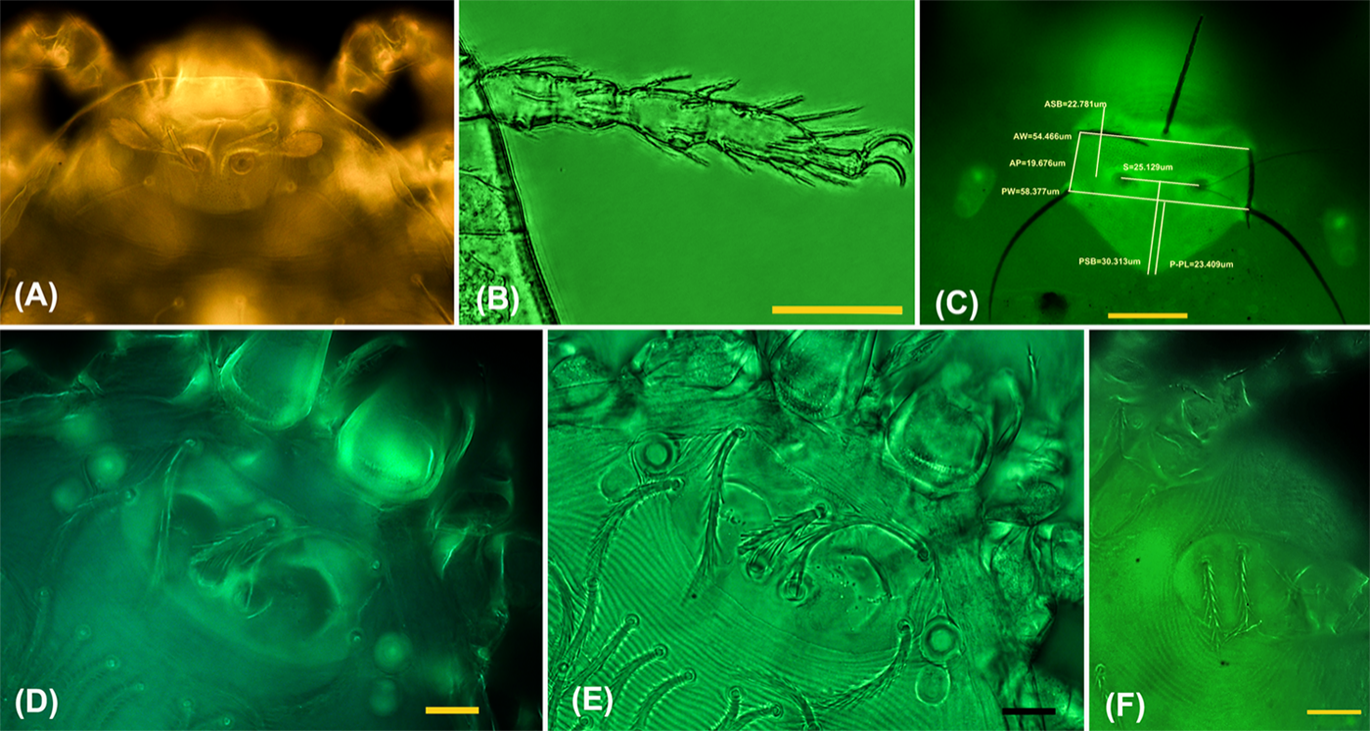
Fluorescence microscopy for trombiculid mite identification. (A) UV light imaging (no filter) with distinct yellow-orange autofluorescence of the trombiculid mite dorsal scutum. (B) Characteristics of setae or claw structures are more delineated using multilayer bright-field imaging with a FITC filter where multiple composite images are combined into one; *Walchia ewingi lupella* leg III (scale bar 35 μm). (C) Autofluorescence (AF) imaging with a FITC filter provides clear scutum images of high resolution, ideal for measurements. Note the prominently fluorescing double eyes; *Blankaartia acuscutellaris* (scale bar 35 μm). (D) Comparison of AF and bright-field (BF) images with FITC filter of the same specimen by switching light-mode; morphological scutum details and setae insertions are rendered more precisely by AF alone, while in panel (E) setae, legs and gnathosome details are sharper when AF is combined with BF illumination, example *Helenicula* sp. (scale bar 10 μm). (F) The usually difficult-to-see setae on coxa III are clearly visible using AF-BF microscopy with FITC filter (scale bar 10 μm).

The images produced with the FITC (green) filter were unequivocally the clearest images of all filters evaluated; 4', 6-diamidino-2-phenylindole (DAPI, blue), APC (allophycocyanin, it can be used as dye that emits red fluorescence), and TexasRed (red). The AF images obtained via the FITC filter clearly enhanced the outline, shape, and details of important features like the scutum, eyes, and setae insertions on the idiosoma and coxae I, II and III, which could barely be identified under BF microscopy (Fig 2, Table 2).

**Table 2:** Mite characteristics requiring autofluorescence (AF) or bright-field (BF) based imaging.

**Mite preparation for microscopy**		
The mite specimen is positioned between two cover slips mounted with distilled water, and placed on a microscope glass slide. The double cover slip approach facilitates turning for ventral and dorsal imaging. All microscopy and imaging is performed under FITC channel.		
**Epifluorescence**	**Normal light / Bright-field microscopy**	
*visible light from above*	*visible light from below*	
single image; one characteristic in optimal focus	single image; one characteristic in optimal focus	multilayer image; multiple images with multiple foci are combined into one
**Scutum**	**Legs (Coxa I to III)**	**Legs (I to III)**
shape and size measurements	coxa I to III setae (base or insertion)	Multilayer images of leg including femur femur (basifemur and telofemur), genu, tibia and tarsus segment
setae/sensilla length, shape and arrangement		Leg I: claws, empodium, pretarsala, subterminala, parasubterminala, microtarsala, tarsala, microtibiala, tibialae, genualae and microgenuala
scutum pitting, pigmentation	Gnathosoma; galeal setae	Leg II; claws, empodium, pretarsala, microtarsala, tarsala, tibialae, genuala
striate or punctuate patterns eyes	galeal setae (branched or nude)	Leg III; claws, empodium, mastitarsalae, mastitibiala, tibiala, genuala and mastifemorala
**Idiosoma; dorsal and ventral overview**	**Scutum**	**Gnathosoma; palps**
setae insertions are enhanced (advantage if setae are damaged or lost)	setae/sensilla length, shape and arrangement	2- or 3-pronged palpal claws
striate or punctuate patterns on coxa	scutum pitting, pigmentation	setae on palps
arrangement of setae on the idiosoma		
	**All setae**	**Gnathosoma; chelicerae**
**Chigger idiosoma**	**length measurement**	**cheliceral blade’s teeth, serration, length**
dorsal overview	idiosoma, scutum or legs	Note: the use of multilayer images is relevant and required for legs (not coxa), palps and chelicerae
	barbed or nude	

**Fig 3:**
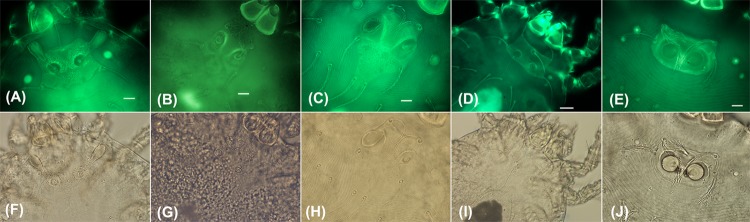
Comparison of autofluorescence (top panels) and bright-field (bottom panels) microscopy of the chigger mite scutum. Fluorescence microscopy enables enhanced visualization of morphological outline, shape and details such as setae insertion points of the scuta. Images represent *Ascoschoengastia* sp. (A, F), *Walchia* sp. (B, G), *Schoengastiella* sp. (C, H) *Leptotrombidium* sp. (D, I), and *Helenicula* sp. (E, J).

**Fig 4:**
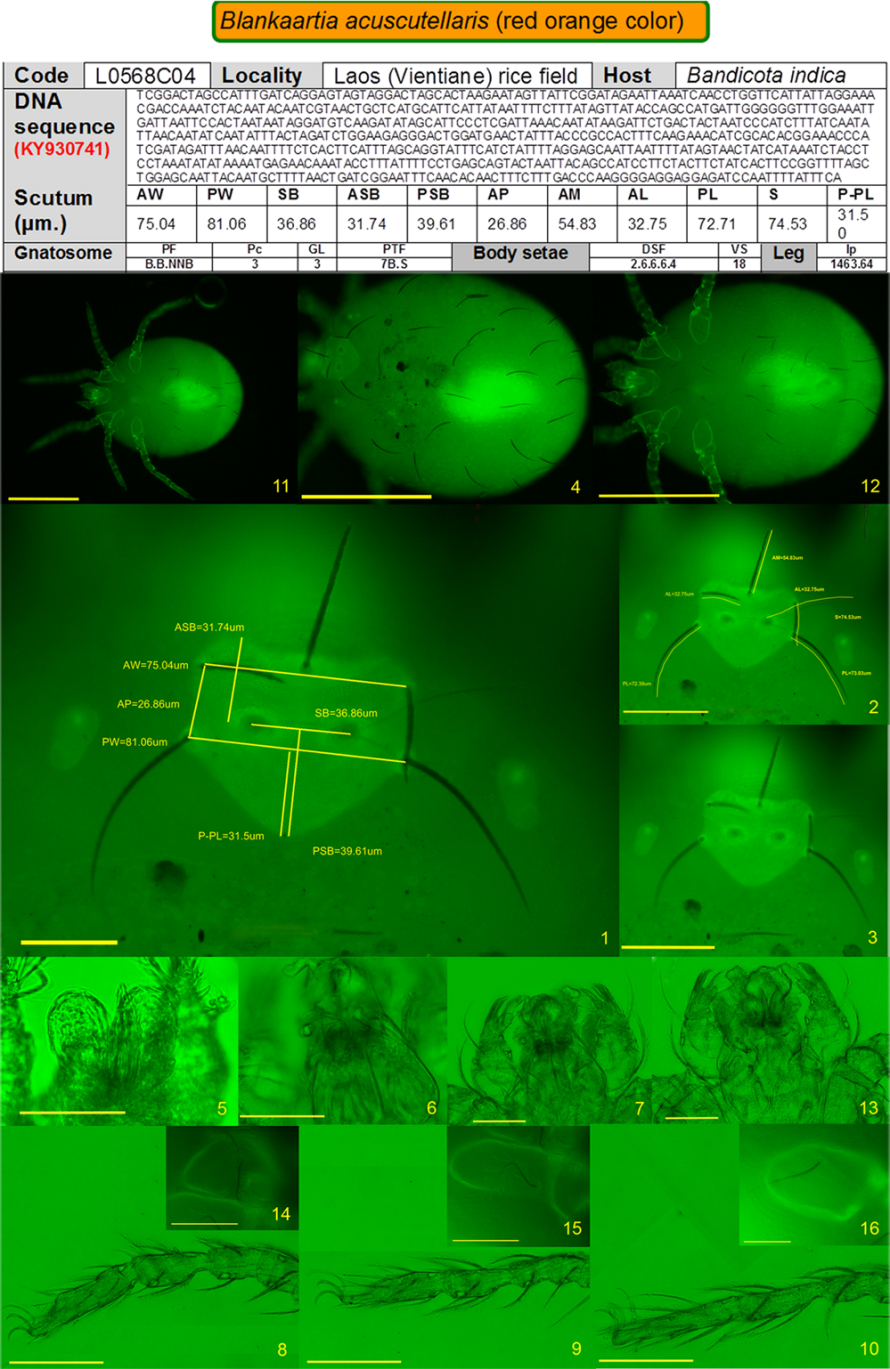
Template of 16 images used to document morphological characteristics prior to genotyping of the specimen. The different panels depict the following: 1. Scutum shape (scale 35 μm); 2. Scutum details (scale 70 μm); 3. Eyes (scale 70 μm); 4. Dorsal idiosomal setae (scale 70 μm); 5. Chelicerae (scale 35 μm); 6. Galeal setae (scale 35 μm); 7. Dorsal view of palps (scale 35 μm); 8–10. Legs I-III (scale 70 μm); 11. Ventral view of idiosoma (scale 350 μm); 12. Ventral idiosomal setae (scale 70 μm); 13. Ventral view of palps (scale 35 μm); 14–16. Coxae I-III (scale 35 μm).

Using AF-BF, the image quality of other characteristics, such as the gnathosoma, scutal setae, sensilla, idiosomal setae and legs, were comparable to images produced by conventional BF microscopy and Hoyer/Berlese-prepared sample specimens. The AF-BF imaging proved superior in delineating fine details, like punctuation, striation or pitting of the scutum, body integument or coxae, as well as setal characteristics (nude or barbed). The AF-BF combination imaging on our defined panel of characteristics substantially facilitated morphological measurement and high-quality imaging of key identifying features (Figs 3 and 4).

### Chigger mite imaging checklist

Based on the above mentioned identification keys for trombiculid mites, a set of 16 images were identified as minimum requirement to enable reliable and complete chigger mite identification to the species level [9–11, 20, 21, 24]. Although some species can be identified with fewer images than 16 due to their distinct and unique features (i.e. *Walchia* spp.), the full identification (and importantly documentation) of uncharacterized chigger mite specimens requires the minimum amount of images as for the species requiring the most images (i.e., *Leptotrombidium* spp.) to enable retrospective, unequivocal identification after the specimen is destroyed for molecular analyses. Hence, a panel of 16 images was developed.

If the chigger features are accurately imaged, the data provided by these 16 images are sufficient to fulfill the requirements of current available identification keys for exact identification of the specimen. The panel of images includes 10 images of the dorsal side [AF and AF-BF for scutum shape/details/measurements, eyes and idiosoma overview; BF imaging for galeal setae; and ML-BF for chelicerae, palps and legs], and six images of the ventral side [AF for idiosoma, ML-BF for palps, and AF-BF coxal setae]. A checklist was developed and validated to cover the features of the currently known spectrum of chigger mites and provide sufficient morphological data to pair with the subsequent genotyping; but if a new species was found and its features required detailed description, then a permanent mounted slide using Berlese or Hoyer medium remained the ideal approach (Fig 1, S1 Table).

### Chigger genus and species identification

The set of 153 permanently prepared chiggers via the Berlese method were re-examined using AF and AF-BF imaging. Exact morphological identification was made to the genus levels, using AF scutum measurements (S2 Fig). The results were unambiguous in all cases, and no diagnostic differences were found when compared to the conventional, accepted reference method. The subgenera characterized by this dataset included *Leptotrombidium*, *Ascoschoengastia*, *Helenicula*, *Schoengastiella* and *Walchia*.

The set of 113 chiggers from Lao PDR was examined by AF and AF-BF imaging for species identification based on the required features and measurements stated in the identification keys by Stekolnikov (2013) [11]. The set of 16 images from our checklist provided sufficient evidence for retrospective evaluation of mites by different experts (Fig 1, S1 Table). All chigger specimens examined could be identified with unambiguous differentiation to the species level.

### Imaging checklist–workflow

Using the proposed workflow, as detailed in the checklist (Fig 1), we rapidly acquired images without spending much time for morphological identification. Even if paired phenotype and genotype data were desired, not all morphological characteristics required immediate analysis, as this could be done post-DNA sequencing. After an initial training phase, the imaging sequence of this workflow became efficient, and a rapid and productive throughput was achieved. The image panel was developed for retrospective characterization of mites to the species level once the genotype was successfully established. The documented image series facilitated second opinions in disputable cases and provided an excellent basis for establishing a database for paired matched genotype and morphotype data of characterized chigger mites (Fig 4).

Multilayer composite imaging (ML-BF) was helpful to enumerate and describe leg setae or appendages. While this was relatively straightforward at a live microscope, where the focus can be adjusted during observation, it was difficult to capture images with focus in different planes that recorded these structures accurately. The acquisition of multilayer images for complicated structures like legs, palpi, or chelicerae can be easily achieved with software. Thus, by combining multiple images into a single stack during post-processing, akin to confocal microscopy, the key details were rendered clearly in a single image with a larger depth-of-field (S3 Fig).

### Genotyping

**Fig 5:**
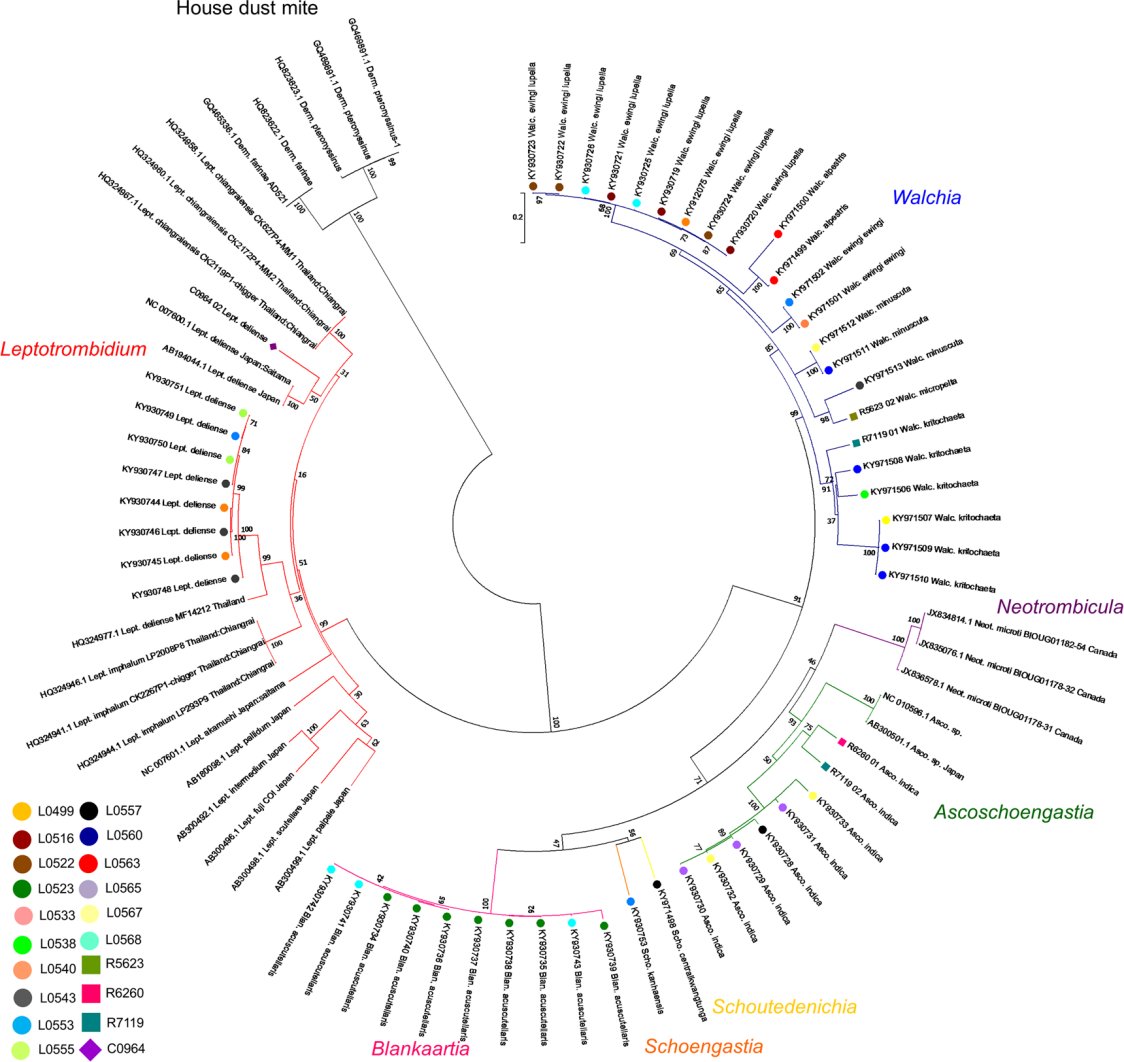
Phylogenetic tree of all currently available *coi* gene sequences of morphotyped trombiculid mites (n = 52 new; n = 25 from GenBank). This study provided 52 new *coi* gene sequences (marked by *, approx. 640 bp length) from Lao PDR (n = 47), Thailand (n = 4), Cambodia (n = 1), and included all available *coi* sequences from NCBI (n = 25). The phylogenetic tree constructed from these *coi* gene sequences demonstrated distinct grouping of assigned morphotypes at the genus levels. Although evidence of both genetic and morphological plasticity was found and sample sizes for each species were small, there was preliminary evidence of sub-structuring of chigger populations below the species level. Different branch colors indicate morphological classification of Trombiculidae [25]; blue = *Walchia*, purple = *Neotrombicula*, green = *Ascoschoengastia*, yellow = *Schoutedenichia*, orange = *Schoengastia*, pink = *Blankaartia*, red = *Leptotrombidium*. Black branch represents sequences of house dust mites (out group). This indicated that DNA extracted from specimens used for autofluorescence analysis was of sufficient quality for downstream PCR amplification.

In this study, successful genotyping was achieved in 52/131 (39.7%) of the AF morphotyped chiggers. In total, we collated 77 *coi* gene sequences from Trombiculidae in Lao PDR (Table 1): 52 from this study (47 from Lao PDR, 4 from Thailand, and 1 from Cambodia) and 25 publically available sequences downloaded from NCBI. The latter comprised sequences from 17 Trombiculid taxa (as of 06 July 2017), included in this research: *Leptotrombidium chiangraiensis* (n = 3), *L*. *imphalum* (n = 3), *L*. *deliense* (n = 3), *Neotrombicula microti* (n = 3), *Ascoschoengastia* (n = 2) and other *Leptotrombidium* taxa (n = 6). The newly obtained sequences were from seven genera: *Leptotrombidium* (n = 9), *Ascoschoengastia* (n = 8), *Walchia* (n = 23), *Blankaartia* (n = 10), *Schoengastia* (n = 1) and *Schoutedenichia* (n = 1), (Table 1 and Fig 5).

### Phylogenetic analysis

The phylogenetic tree constructed from these *coi* gene sequences (approx. 640 bp in length) demonstrated distinct grouping of assigned morphotypes at the genus and subgenus levels (Fig 5). Generally the *coi* sequences from our Lao PDR mites clustered according to the assigned morphotypes, while previous *coi* sequences from mites downloaded from GenBank were clearly separated. The *L*. *deliense* sequences from Lao PDR grouped distinctly from previously deposited sequences from northern Thailand, and the single sequence from Nan Province was also separated. However, evidence for intra-cluster variation was observed even within mites collected from the same rodents. *L*. *chiangraiensis*, *L*. *akamushi*, and *L*. *imphalum* were unambiguously grouped, as they did not mix within other *Leptotrombidium* sequences. The *Walchia* sequences demonstrated the most pronounced genetic variation, with distinct separation of the *W*. *kritochaeta*, *W*. *alpestris*, *W*. *minuscuta*, *W*. *ewingi ewingi* and *W*. *ewingi lupella* groups. Interestingly, the northern Thailand derived *W*. *kritochaeta* grouped within the *W*. *kritochaeta* group from Lao PDR. The *W*. *alpestris* mites collected from the same rodent showed substantial *coi* gene divergence, while *W*. *ewingi lupella* showed the most pronounced variation. The *Ascoschoengastia* sequences clustered in the same group with *Ascoschoengastia* spp. from the NCBI data. The *Blankaartia* sequences grouped in a single cluster, but demonstrated some intra-cluster variation between mites derived from a single rodent (ID KY930734 to KY930743 in Fig 5).

## Discussion

This study has demonstrated that a combination of AF and BF microscopy imaging can provide sufficient resolution and information to characterize trombiculid mites to the species level, while preserving the mite genomic DNA for subsequent genotyping of the same individual chigger.

The method takes advantage of the autofluorescent properties of the exoskeleton of some chelicerate species for mite identification. The morphological criteria of previously defined keys for trombiculid mites were reliably fulfilled using AF, BF or AF-BF in 113 mites of 7 genera to the species level (using a set of 16 images), and in 153 mites to the genus level.

The specimen preparation for autofluorescence imaging is simple and straightforward requiring only that the sample is mounted in a drop of sterile water (or ethanol) and sandwiched between coverslips for ease of imaging both ventral and dorsal aspects. Mounting in ethanol or water will not affect the subsequent DNA extraction yield in quality or quantity. The advantage of characterizing the chigger specimen without using any clearing agent that could affect the genetic material is significant, as it will enable cross-validation between morphological and genetic typing, providing paired-matched data, which can be prospectively collected across various geographical regions to provide a representative database for subsequent molecular taxonomic studies.

### Autofluorescence for microscopy

The autofluorescent properties of arthropods, including mites, are most pronounced under UV light; *i*.*e*., electromagnetic radiation of wavelengths shorter than visible light (10 nm-400 nm). However, DNA damage with fragmentation and dimer formation is associated with UV light, and caused primarily by UV-B (280–315 nm). UV-induced DNA damage results in DNA strand breaks impacting genome integrity [26]. The mercury arc lamps emit light across a range of peaks in the UVC, UVA and visible range (184–578nm). Using a FITC filter narrows the radiation range to an excitation wavelength of 490 nm, which is blue visible light that has no UV component to damage the DNA of a specimen (fluorescein has an absorption peak at 494 nm and emission peak at 512 nm). We found that the autofluorescence properties of the chitin skeleton of arthropods are clearly visible using a FITC filter, without affecting the specimen DNA, and hence based our imaging on this light source. The use of epifluoresence microscopy with a FITC filter actually improved image quality and contrast compared with that obtained using conventionally cleared specimens and BF microscopy, and provided excellent results in the blue/green spectrum of light, enabling long exposure without danger of DNA damage and/or bleaching issues. This technique resembles SEM, as it provides high-resolution scanning of the mite surface, without any penetration into the body as would be the case with diaphanoscopy or transillumination techniques. The autofluorescence intensity increases with the thickness of the chitin layer under scrutiny, which is why the dorsal scutum stands out clearly and the differential fluorescence intensity can be utilized for sharp fluorescence imaging (Fig 2).

Several clearing-mounting agents have been used in arthropod specimen preparation, e.g., Hoyer’s medium and Berlese fluid. These media contain the same vital ingredients: aqueous gum-chloral (chloral hydrate) and glycerol, which macerate both soft tissue and external sclerotized chitinous parts of arthropods [17, 27, 28] Although glycerin exhibits DNA preservation properties, the combination with chloral hydrate, a genotoxic substance, inevitably leads to DNA damage in the preparations, impairing gene sequencing from DNA extracted material [29–32].

### Imaging checklist–a novel workflow

Autofluorescence imaging exhibited excellent images of the scutum and idiosomal setae arrangement, which provided enough information to identify chiggers to the subgenus level, while for some characteristics of legs and gnathosome, the use of BF and/or AF-BF light significantly improved imaging (Figs 2 and 3, Table 2). AF-BF imaging consistently allowed at least equivalent, or improved, unambiguous identification of all features normally seen in the previous Hoyer-based preparation methods (Fig 3).

The proposed workflow worked effectively using 16 images for morphological identification to the genus and the species levels, respectively–and proved to be rapid once a routine was established, including acquisition of multilayer composite imaging of legs, palpi, and chelicerae. However, for description of new species, the advantages of a permanently mounted specimen for repeated and detailed scrutiny became evident. This is because static imaging cannot provide the level of information afforded by constant refocusing and repositioning of particular mite features. Hence, mounting medium preparations will continue to be required for the description of novel species until digital 3D imaging becomes available. Nevertheless, the proposed new workflow will contribute substantially to future expansion of high quality paired morphological and genotypic data for trombiculid mites.

### Paired morphological and molecular mite ID–benefits and implications for a genetic database

Mite identification based on paired morphological and molecular ID will contribute to and facilitate the prospective expansion of a reference database of *coi* gene sequences (and 18S rDNA sequences). Such a database will facilitate molecular ID without the fastidious morphotyping in the future as it will support BLAST search/analyses against previously characterized sequences, enable more in-depth investigations into aspects of genetic and morphometric variation among mites, and provide a basis on which to unravel associations between mites, their mammal hosts, their endosymbionts and pathogens, and possibly with the human dead-end hosts—especially in the case of scrub typhus transmission.

One problem hindering the correct molecular barcoding is the presence of nuclear mitochondrial pseudogenes (numts), which might be co-amplified with their orthologous mitochondrial DNA, resulting in paralogous sequences and vagueness in DNA barcoding [33]. A previous study attempting to characterize mitochondrial gene content and arrangement in chigger mites revealed that a pseudogene of the small subunit rRNA was found in *Leptotrombidium pallidum*, but not in the other three *Leptotrombidium* species studied (*L*. *akamushi*, *L*. *deliense*, and *L*. *fletcheri*) [34]. Several attempts to control numts include utilization of RT-PCR or long PCR, mitochondrial DNA enrichment [35], or amplification of longer fragments (numts usually have a shorter length of less than 700 bp) [36]. In our study, we initially amplified a 710 bp fragment of *coi* gene, before cropping it to an equal size of around 640 bp. We removed sequences with ambiguous double peaks, and used the ExPASy, Bioinformatics Resource Portal (translation tool) to identify open reading frames (ORFs) and stop codons. Then, we aligned our sample sequences with other known DNA sequences in NCBI database. With these efforts, we attempted to minimize the ambiguity due to numts during DNA barcoding.

Currently, there is very little known about the morphological features of adult trombiculid mites and no useful identification charts are available. The *coi* gene sequence should remain unchanged throughout the mite cycle and once robust morphological features (currently of the larval stage) are paired to a genotype, the genetic information will assist in further morphological identification of other stages of the mite. Thus, characteristics documented for deutonymphs or adult stages could be subsequently assigned to a previously validated molecular ID for pairing/matching.

The prospective acquisition of phenotypically-paired molecular identifications for chiggers from various geographical regions will result in a broad representative spectrum of mite genera and species that will enable linkage with topography, habitats, biodiversity of fauna and host species. Large scale attempts have been made using morphotyping alone–and a reliable method for producing paired morphological and genetic data is now required to move the field ahead [37]. It must be emphasized that all molecular identifications are only as good as the phenotypic data on which they were originally based. Until there is a large *coi* database available from reliably morphotyped chiggers, molecular barcoding cannot begin to replace the painstaking morphological work.

### Molecular identification of trombiculid mites

A previous report of chiggers with phenotypic data from Korea was based on the 18S rDNA gene, but no genetic variation was seen in 38 reported chigger sequences [38]. The available data on *coi* genes for trombiculid mites on GenBank (n = 25) was put into context when our new sequences (n = 52) were added into the phylogenetic analysis (Fig 5). Although sample sizes for each species were small, there was preliminary evidence of sub-structuring of chigger populations below the species level. Evidence of both genetic polymorphism and morphological plasticity within species was found, especially within the *Ascoschoengastia* and *Walchia* genera. For example, *Walchia ewingi lupella* mites derived from *Bandicota indica* rodents actually demonstrated genetic diversity within a cluster of mites obtained from a single location on a single host, with high bootstrap values. This variation may reflect the natural transient carriage on rodents during feeding within or between mite “islands”, leading to breeding between different populations within a species of mites that may be partially reproductively isolated.

Interestingly, we also observed substantial morphological variation within the *Walchia* genus, related to morphometric data of the scuta. A recent study revealed that morphological differences in the chigger mite *Hirsutiella zachvatkini* (Schluger) were host-associated and related to *Apodemus agrarius*, *Apodemus flavicollis* and *Myodes glareolus* rodent carriage [39]. If the morphological variation observed in this study relates to prolonged attachment to certain rodents and can be attributed to phenotypic plasticity–in a similar way as recently observed in *Hirsutiella zachvatkini*—remains to be determined by future work [40]. Clearly, there is much to be learned on the intraspecific variation of morphometric characteristics, which—supported by molecular genotyping–needs to be more comprehensively investigated for the members of the Trombiculidae family.

The clustering patterns of Trombiculidae *coi* sequences revealed the 5 major subclades *Walchia*, *Ascoschoengastia*, *Blankaartia*, *Neotrombicula and Leptotrombidium* with high discriminatory power supporting genus identification (Fig 5). Special care is required for removing host tissue residues attached to chigger mouth parts, as these could lead to host-related amplicons from chigger DNA preparations.

### Molecular chigger identification and creation of a database

The published PCR primers for the *coi* gene were previously shown to successfully amplify across a wide diversity of invertebrates, and worked well on trombiculid mites. However, the underlying reasons for the low success rate in determining a molecular barcode for previously morphotyped chiggers remain unclear–this was 40% in our study and 50% in a previous study in Korea [38]. It is possible that chigger DNA could degrade in 95% ethanol-preserved specimens, due to incomplete or slow penetration of ethanol through the arthropod cuticle leading to enzymatic degradation of DNA within the organs and hemocoel. Further, the need to rehydrate and wash in pure water may also contribute to degradation, due to the low pH of pure water, and both factors could contribute to the high *coi* gene PCR and sequencing failure rates. Optimization of fixation procedures should be prioritized, as the likelihood of suboptimal PCR primer design is low. However, as more gene sequences become available and more genotypes are matched with morphological criteria, improved PCR assays can be developed in the future.

### Relevance for scrub typhus research

Recently, Park *et al*. collected chigger mites from urban areas in Seoul, Korea, and using phenotypic and genetic criteria (18S rDNA sequencing), linked them to *Orientia tsutsugamushi* infection in humans and carriage in mites, suggestive of transmission [38]. The possibility of identifying the vectors of a pathogen and linking both to clinical cases caused by this pathogen would greatly enhance our understanding of the natural history of scrub typhus, shed light on the role of different vectors and strains, especially in geographic areas considered non-endemic and would support approaches to reduce morbidity and mortality [41–44]. The potential of this approach is considerable: if mites and their endosymbionts are characterized within endemic areas where humans acquire the disease, then the genetic diversity and population genetics of *Orientia tsutsugamushi* could be better understood. These findings could translate into disease prevention, inform on the pathogenicity of *Orientia* spp., elucidate pathogen-vector associations and the distribution of vectors and their hosts, as well as allowing characterization of the phenotypic and genetic variation of *Orientia* spp.-harboring mites [45].

We conclude that combining autofluorescence with bright field microscopy for morphotyping of chiggers provides at least as good, if not improved, microscopic imaging than the previous methods involving mite maceration using Berlese and/or Hoyer’s media for mounting preparation. The substantial benefit of this method lies in the preservation of mite DNA for subsequent genotyping, and the potential of providing paired morphotype and genotype data for each individual chigger—with a single study we have tripled the available characterized *coi* gene sequences on GenBank. This method will be of major importance to create a future database where *coi* sequences can be accessed for rapid trombiculid mite identification, which will support epidemiological, genetic and ecology-related investigations.

## Supporting information

S1 Fig Chigger mite colonies on rodent ears. (A) Chigger mites infested on rodent ear. (B)-(D) Remark of chigger mites position selecting Images of rodent ears with chigger mites were taken using a stereo to mark exact chigger mite locations from colonies on the rodent ear, which were selected by purposive sampling for broad representative coverage. Panel A: Overview image. Same ear with details on specific areas with multiple chiggers feeding–chigger species are labeled to highlight variation in size and color (panels B-D).
(TIF)

S2 Fig Panel of autofluorescent mite scutum images demonstrating the morphometric features of five genera of trombiculid mites. The set of 153 permanently prepared chiggers via the Berlese method were re-examined using AF and AF-BF imaging, and exact morphological identification was made to the genus levels, using AF scutum measurements (5 genera shown, 7 specimens each, 35 in total). The subgenera characterized by this dataset included *Leptotrombidium*, *Ascoschoengastia*, *Helenicula*, *Schoengastiella* and *Walchia*.
(TIF)

S3 Fig Multilayer composite imaging–using bright-field phase contrast microscopy for mite identification. Panel A: Composite image of seven layers of the *W*. *ewingi lupella* leg III, with inserted panels A1-A3 depicting individual layer images focused on different setae (all scale bars 35 μm). While independent images appear out of focus, the stacked multilayer composite image is sharp, revealing more detail and depth-of-field than one single image. Panel B: Schematic diagram of the legs I, II and III (leg I on top) demonstrating the general segments, special setae and details of the terminalia, tarsalia and claws (37).
(TIF)

S1 Table Checklist of required images for mite characterization. (DOCX)
