# Supplementary figures and images for: Autofluorescence microscopy for paired-matched morphological and molecular identification of individual chigger mites (Acari: Trombiculidae), the vectors of scrub typhus
Source: PLoS One. 2018 Mar 1;13(3):e0193163. doi: 10.1371/journal.pone.0193163 (PMC5832206; doi:10.1371/journal.pone.0193163)

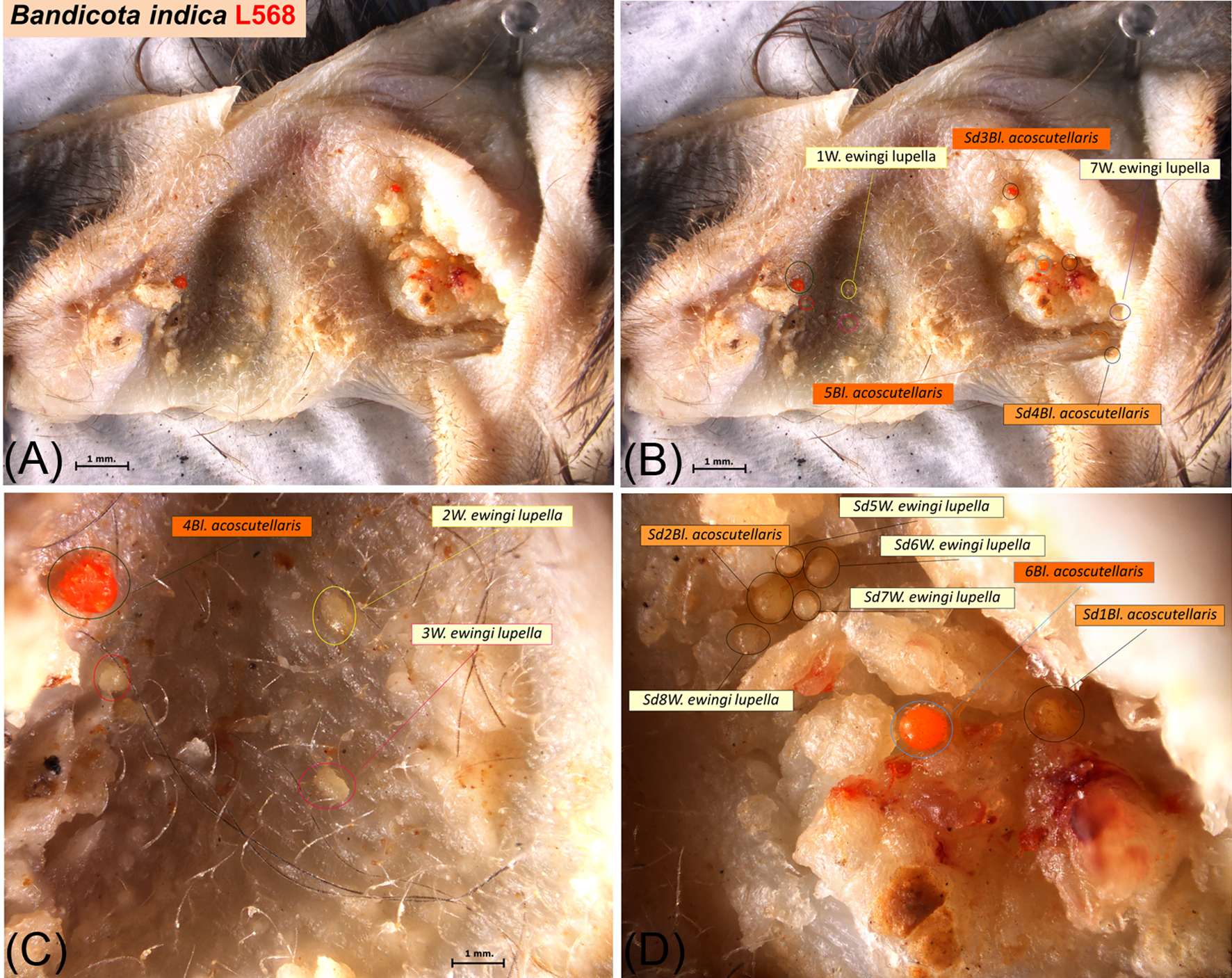

Supplement: S1 Fig — (A) Chigger mites infested on rodent ear. (B)-(D) Remark of chigger mites position selecting Images of rodent ears with chigger mites were taken using a stereo to mark exact chigger mite locations from colonies on the rodent ear, which were selected by purposive sampling for broad representative coverage. Panel A: Overview image. Same ear with details on specific areas with multiple chiggers feeding–chigger species are labeled to highlight variation in size and color (panels B-D). (TIF) [file pone.0193163.s001.tif]

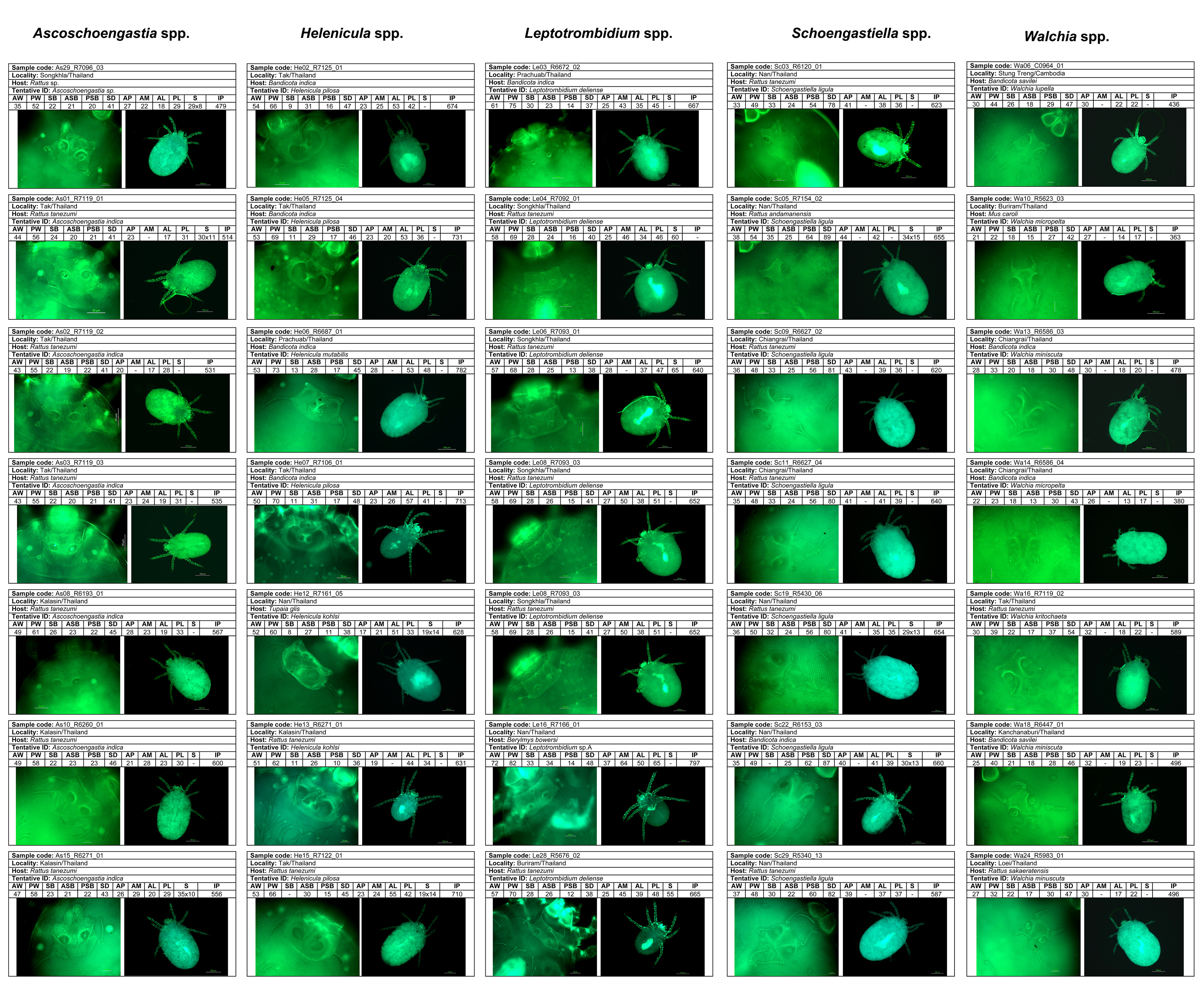

Supplement: S2 Fig — The set of 153 permanently prepared chiggers via the Berlese method were re-examined using AF and AF-BF imaging, and exact morphological identification was made to the genus levels, using AF scutum measurements (5 genera shown, 7 specimens each, 35 in total). The subgenera characterized by this dataset included Leptotrombidium, Ascoschoengastia, Helenicula, Schoengastiella and Walchia. (TIF) [file pone.0193163.s002.tif]

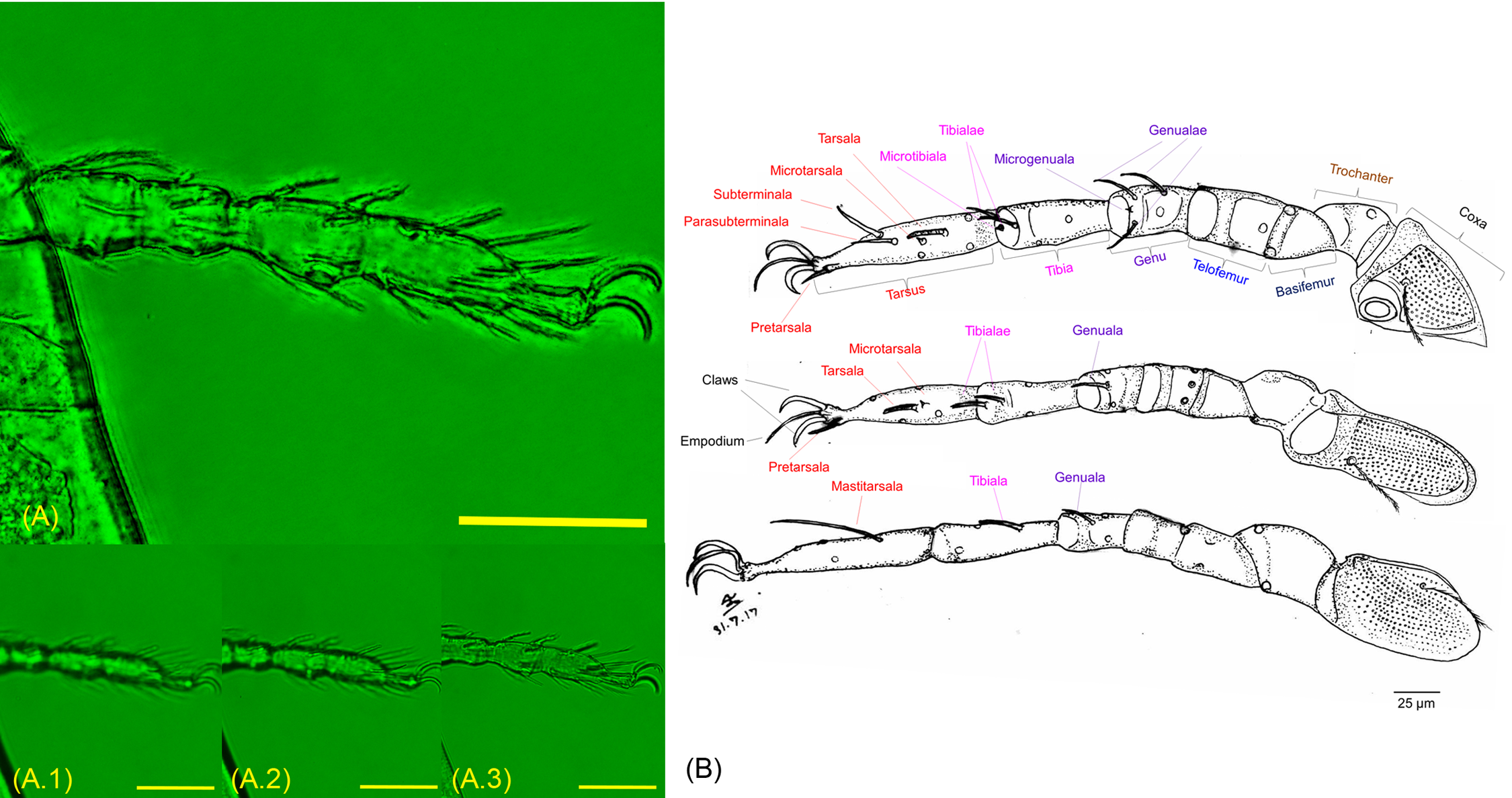

Supplement: S3 Fig — Panel A: Composite image of seven layers of the W. ewingi lupella leg III, with inserted panels A1-A3 depicting individual layer images focused on different setae (all scale bars 35 μm). While independent images appear out of focus, the stacked multilayer composite image is sharp, revealing more detail and depth-of-field than one single image. Panel B: Schematic diagram of the legs I, II and III (leg I on top) demonstrating the general segments, special setae and details of the terminalia, tarsalia and claws (37). (TIF) [file pone.0193163.s003.tif]
