# Supplementary material for: Autofluorescence microscopy for paired-matched morphological and molecular identification of individual chigger mites (Acari: Trombiculidae), the vectors of scrub typhus
Source: PLoS One. 2018 Mar 1;13(3):e0193163. doi: 10.1371/journal.pone.0193163 (PMC5832206; doi:10.1371/journal.pone.0193163)
Supplement: S1 Table — (DOCX) [file pone.0193163.s004.docx]

| **Checklist for morphotyping. Mite description / location / Code No.**  *Note: all images with FITC filter – fluorescence (AF) or bright-field (BF) – Multilayer (ML)* | | | | | |
| --- | --- | --- | --- | --- | --- |
| **No.** |  | **Region** | **Description** | **Type** | **Check** |
|  |  | **Dorsal images** |  |  |  |
| **1** | 40x | **Scutum shape** | Scutum whole contour, setae insertion points (morphometrics) and pigmentation | AF |  |
| **2** | 40x | **Scutum details** | Focus on setae, sensilla and pigmentation | AF/BF |  |
| **3** | 40x | **Eyes** | Focus on eyes – single or double pairs | AF |  |
| **4** | 40x | **Dorsal idiosomal setae** | Dorsal setae arrangement (for engorged chiggers acquire 4 quarter images) | AF |  |
| **5** | 40x | **Chelicerae** | Chelicerae detail – blade with tricuspid cap | ML-BF |  |
| **6** | 40x | **Galeal setae** | Galeal setae – nude or branched | BF |  |
| **7** | 40x | **Dorsal view of palps** | Dorsal view of palps - focus on femoral, genual, dorsal, lateral and ventral tibial setae, prongs of palpal claw | ML-BF |  |
| **8** | 40x | **Legs I** | Dorsal - focus on claws, empodium and specialized setae | ML-BF |  |
| **9** | 40x | **Legs II** | Dorsal – focus on claws, empodium and specialized setae | ML-BF |  |
| **10** | 40x | **Legs III** | Dorsal – focus on claws, empodium and specialized setae | ML-BF |  |
|  |  | **Ventral images** |  |  |  |
| **11** | 10x | **Ventral view of idiosoma** | Whole ventral overview of idiosoma | AF |  |
| **12** | 40x | **Ventral idiosomal setae** | Ventral setae distribution *(for engorged chiggers acquire 4 quarter images)* | AF |  |
| **13** | 40x | **Ventral view of palps** | Ventral view of palps - focus on ventral tibial and tarsal setae | ML-BF |  |
| **14** | 40x | **Coxa I** | Ventral - focus on base setae insertion(s) Coxa I | AF-BF |  |
| **15** | 40x | **Coxa II** | Ventral - focus on base setae insertion(s) Coxa II | AF-BF |  |
| **16** | 40x | **Coxa III** | Ventral - focus on base setae insertion(s) Coxa III | AF-BF |  |
